# In Vitro and In Silico Analysis of the Inhibitory Activity of EGCG-Stearate against Herpes Simplex Virus-2

**DOI:** 10.3390/microorganisms10071462

**Published:** 2022-07-20

**Authors:** James D. Stamos, Lee H. Lee, Calvin Taylor, Tony Elias, Sandra D. Adams

**Affiliations:** Biology Department, Montclair State University, Montclair, NJ 07043, USA; james.stamos@nih.gov (J.D.S.); leel@montclair.edu (L.H.L.); taylorc13@montclair.edu (C.T.); eliast89@rowan.edu (T.E.)

**Keywords:** HSV-2, EGCG, EGCG-stearate, antiviral, qPCR, bioinformatics

## Abstract

About half a billion people worldwide are infected with herpes simplex virus-2 (HSV-2). Prolonged treatment with acyclovir (ACV) and its analogs leads to the development of resistant strains. The aim of this study was to investigate the antiviral potential of epigallocatechin gallate (EGCG) from *Camellia sinensis* and a stable analog EGCG-stearate (EGCG-S) against HSV-2 in cultured Vero cells. Cell viability and cell proliferation assays were used to determine the non-cytotoxic concentrations on cultured Vero cells. HSV-2 with a green fluorescent protein (GFP) fusion protein of VP26 virions were treated with non-cytotoxic concentrations of EGCG and EGCG-S. The effects on infectivity and mechanisms were determined by plaque assay, attachment and penetration assays, confocal microscopy, qPCR, and in silico modeling analysis. Our results demonstrate that treatment of HSV-2 virions with EGCG and EGCG-S at a concentration of 75 µM showed greater than 99.9% inhibition by inhibiting the attachment of HSV-2 virions to host cells. The bioinformatic analysis indicated high binding affinity of EGCG-S for glycoprotein D; thus EGCG-S may block fusion of HSV-2 and the cell membrane, preventing entry of HSV-2 into the cell.

## 1. Introduction

Herpes simplex virus type 2 (HSV-2) is a member of the family *Herpesviridae*, subfamily *Alphaherpesvirinae* [1]. HSV-2 virions consist of an inner core with linear, double-stranded DNA that is enclosed in a capsid. An outer envelope containing various glycoproteins covers tegument proteins, which are exterior to the viral capsid [2]. Both attachment and penetration take place when viral glycoproteins bind to cellular receptors on the plasma membrane of the host cell [3,4]. Adsorption requires participation from multiple viral glycoproteins and cellular receptors [5]. Because these viruses enter the latent phase in sensory ganglia [6], the immune system fails to clear the infection, and this results in recurrent genital lesions. Thus, treatments that reduce the severity and duration of these sores are highly desirable.

Acyclovir (ACV), famciclovir, and valacyclovir are among the most effective antiviral medications that exist for HSV infections [7]. ACV selectively inhibits HSV DNA polymerase and prevents elongation [7]. Limitations of the use of ACV included limited solubility in water, short half-life in the blood, and poor oral bioavailability. This resulted in the need for an increased dosage to remain effective, thus increasing toxicity. Prolonged use of these medications could result in the formation of new resistant strains of HSV [8]. HSV resistance to ACV is most commonly due to mutations in the viral thymidine kinase gene [9].

Polyphenolic molecules derived from plants have gained popularity recently as potent nontoxic antiviral compounds. Green tea, black tea, and oolong teas derived from the *Camellia sinensis* plant contain polyphenols [10,11,12]. The method of preparation of green tea preserves the major polyphenolic compounds known as catechins. Research has shown that epigallocatechin-3-gallate (EGCG), the most abundant catechin in green tea, has a wide array of therapeutic applications including antioxidant, anti-inflammatory, anti-tumorigenic, and antibacterial [13,14,15,16,17,18,19] applications.

EGCG has been demonstrated to have antiviral properties against several viruses including influenza, HSV-1, human immunodeficiency virus (HIV), Zika, adenovirus, hepatitis B and C, and human coronaviruses including SARS-CoV-2, human coronavirus HCoV-0C43 (beta coronavirus), and HCoV-229E (alpha coronavirus) [20,21,22,23,24,25,26,27,28,29,30]. The mechanism of action determined in several antiviral studies was that EGCG inhibited viral entry [20,21,22,26,28]. A previous study suggested that treatment of HSV virions with EGCG could inactivate virions by forming complexes with viral glycoproteins B and D [27]. Reduced viral yield was demonstrated by the additive effects of EGCG (25 µg/mL) and acyclovir (50 µg/mL) on HSV-1 infection in oral epithelial cells [31]. EGCG, however, is not stable in aqueous solutions and at higher pH [32,33]. EGCG is not stable at physiological pH; therefore it has limited therapeutic potential [34,35]. It has been proposed that lipid-soluble tea polyphenols could improve the effectiveness of these formulations [32,35,36]. Lipophilic modifications of EGCG, palmitoyl-EGCG [37], and EGCG-stearate [38], were found to inhibit HSV-1. A proprietary topical application containing EGCG-stearate (Camellix, LLC, Augusta, GA, USA), was administered to two patients with recurrent HSV-1 infections, reducing the symptoms of inflammation [39].

Our study aimed to investigate the efficacy and mode of action of the more stable structural analog EGCG-stearate (EGCG-S) against HSV-2 in cultured Vero cells.

## 2. Materials and Methods

### 2.1. Cell Culture

Green monkey kidney cells (Vero, ATCC CCL-81; ATCC, Manassas, VA, USA) were cultured in 5% fetal bovine serum (FBS)–DMEM supplemented with 1 µg/mL gentamicin. Cells were maintained in vented flasks kept at 37 °C in 5% CO_2_.

### 2.2. HSV-2 Maintenance

HSV-2 (ATCC VR-1781) (ATCC, Manassas, VA, USA) and HSV-2 VP26-GFP [40] which expresses a fusion protein of VP26 and GFP (generously donated by Dr. Andrea Bertke, Virginia Tech University) were used in all experiments. Preparation of virus was previously described in De Oliveira et al. [37]. Passage of the virus was performed in T-25 flasks, and cells were allowed to reach complete cytopathic effect (CPE). The viral media were then collected, centrifuged, and the supernatants containing viruses were kept in cryogenic vials at −80 °C_._

### 2.3. Preparation of Polyphenols

EGCG was purchased from Camellix, LLC (Augusta, GA, USA) and EGCG-S (US Patent 20120172423) was modified by and purchased from Camellix, LLC (Augusta, GA, USA). Samples were prepared based on the method described in Patel et al. [38]. EGCG and EGCG-S were dissolved in dimethyl sulfoxide (DMSO) to prepare initial 5 mM stock concentrations. All stock solutions were freshly prepared and subjected to filtration prior to application.

### 2.4. Cell Viability

Vero cells were plated in 6-well plates and treated with various concentrations (25, 50 and 75 µM) of EGCG and EGCG-S, DMSO, ethanol, and culture media for one hour. The highest DMSO final concentration (1.5%) was used to test the cytotoxic effect on Vero cells. Cells were harvested 48 h post-treatment. Cells were then stained with trypan blue and counted by using a hemocytometer. Relative cell viability was determined and compared to the Vero cells controls as 100% viable.

Cell viability (%) = (total viable cells (unstained)/total cells (stained and unstained)) × 100%(1)

### 2.5. Cell Proliferation

Vero cells were cultured in 96-well plates for 24 h and treated with appropriate concentrations of polyphenols for one hour. After 24 h, 10 µL of WST-1 reagent (Roche Diagnostics, Indianapolis, IN, USA) were then added for two hours to the samples and the absorbance was read by using a microplate reader with a wavelength of 450 nm. The method was previously described in detail in Cantatore et al. [41]. The WST-1 cell proliferation assay is a colorimetric assay based on the cleavage of a tetrazolium salt by mitochondrial dehydrogenases to form formazan in viable cells. It is used for the measurement of cell proliferation and cell viability (Roche Diagnostics, Indianapolis, IN, USA).

% Proliferation = ((Treated cells − Blank)/(Cells only − Blank)) × 100%(2)

### 2.6. Antiviral Assay

Vero cells were plated in a 96-well plate and after 24 h, HSV-2 was treated with 25 µM, 50 µM, and 75 µM EGCG-S and EGCG. The method, with modifications, was previously described in Patel et al. [38]. After treatment, cells were infected with treated and nontreated virus and incubated for one hour at 37 °C and 5% CO_2_. Any unadsorbed virus was aspirated and replaced with 100 µL of 5% FBS-DMEM media. After 72 h, 10 µL of ToxGlo™ reagent (Promega, Madison, WI, USA) was added to all wells containing samples (controls included 100 µL of 5% FBS DMEM media both with and without the ToxGlo™ reagent), then incubated at 37 °C and 5% CO_2_ for 1 h. The relative light unit (RLU) values of each well were recorded by using the Infinite 2000 PRO microplate reader (Tecan Life Sciences US, Raleigh, NC, USA).

### 2.7. Cell Morphology

Vero cells were plated in 6-well plates and were infected with HSV-2 treated with 75 µM EGCG-S, 75 µM EGCG, or nontreated HSV-2 (MOI = 0.1) for one-hour incubation, with intermittent rocking at 37 °C and 5% CO_2_. Analysis of cell morphology was previously described in De Oliveira et al. [37]. The effect of virus infection was compared to the uninfected Vero cells control. After one hour, the unadsorbed virus was aspirated and 2.5 mL of media was added to each well and incubated at 37 °C and 5% CO_2_ for 48 h. Morphological changes were observed at day 2 post-infection by using an inverted microscope.

### 2.8. Plaque Assay

Vero cells were cultured in 6-well plates until 100% confluent. The virus was treated with 75 µM EGCG or EGCG-S for one hour. The untreated virus served as the control. Virus dilutions ranging from 10^−3^ to 10^−7^ were prepared. Cells were infected with the diluted virus for one hour and the unadsorbed virus was aspirated. Plaque assay method is a modification of protocol previously reported in Adams et al. [42]. After one hour of incubation, cells were overlaid with plaque media consisting of bacteriological agar containing 3X Eagle medium (Gibco Invitrogen Corporation, Grand Island, NY, USA), 1.5 mL of 5% sodium bicarbonate (Gibco Invitrogen Corporation, Carlsbad, CA, USA), 0.5 mL FBS, 0.1 mL DEAE-dextran (ICN Biomedicals Incorporated, Aurora, OH, USA), 0.1 mL penicillin/streptomycin (Cambrex, Walkersville, MD, USA), with 0.05 mL gentamicin and 0.6% bacteriological agar (Oxoid Limited, Baskingstoke, Hampshire, England). Both solutions were placed in a 41 °C water bath, combined 1:1, and cells were overlaid with 3 mL of plaque media.

After 72 h of incubation at 5% CO_2_, cells were stained with crystal violet and plaques were counted followed by the calculation of mean and standard deviation.

% inhibition = [1 − (PFU treated/PFU control)] × 100%(3)

### 2.9. Attachment Assay

Vero cells were grown in a 96-well plate until 70–80% confluent. The plate was pre-incubated at 4 °C for 30 min. Subsequently, the media was removed from the cells. The virus was treated with concentrations of 50 and 75 µM EGCG-S and EGCG, respectively. Treated and untreated HSV-2 virions were incubated at room temperature for one hour followed by infection of the cells on ice. The plate was incubated for 2 h at 4 °C, then unbound HSV-2 was carefully aspirated from the cells [39]. DMEM was added to each well and the plate was incubated at 37 °C and 5% CO_2_ for 48 h and assayed by using the Viral ToxGlo™ assay (Promega). The attachment protocol was adapted as previously described in Cantatore et al. [41] with minor modifications. The mean and SD of 4 replicates were calculated.

### 2.10. Penetration Assay

Vero cells were grown in a 96-well plate until 70–80% confluent. Cells were pre-chilled at 4 °C for 1 h, and then the virus was added (MOI = 0.1) and incubated at 4 ° for 2 h. The protocol was adapted with minor modifications from Cantatore et al. [41]. Concentrations of 50 and 75 µM EGCG-S and EGCG, respectively, were added and the cultures were incubated for 60 min, respectively. Infected cell monolayers were treated with acidic PBS (pH 3) for 1 min to deactivate particles that did not enter, and PBS (pH 11) was immediately added to balance the medium pH [43,44]. DMEM was added to each well and the plate was incubated at 37 °C and 5% CO_2_ for 48 h and assayed by using the Viral ToxGlo™ assay (Promega). Means and SD of 4 replicates were calculated.

### 2.11. Confocal Microscopy

The protocol was described in detail in Patel et al. [38] with minor modifications. Vero cells were grown on glass coverslips within 12-well plates and were infected with treated (75 μM EGCG-S) or non-treated HSV-2 VP26-GFP for one hour (MOI = 0.3). Twelve hours post-infection, cells were stained with 300 µL of 300 nM DAPI (4,6-diamidino-2-phenylindole) for 5 min at 37 °C in the dark. Cells were then fixed with 4% paraformaldehyde solution for 20 min and rinsed with phosphate-buffered saline (PBS). The glass coverslips containing cells were glued to a slide by using a drop of clear nail polish. Cells were then visualized and were examined under a Leica SP5 scanning confocal microscope under 10× or 63×/1.4 NA water Plan Apo objective at Vassar College (Poughkeepsie, NY, USA).

### 2.12. Primer Design and Quantitative Polymerase Chain Reaction (qPCR)

Vero cells grown in 60-mm culture plates, when 70–80% confluent, were infected with treated HSV-2 VP26-GFP. The virus was treated with 75 µM EGCG-S for one hour. Uninfected cells served as a negative control, and untreated virus served as a positive control. After 60 min of infection, the media was aspirated, and new media was added. Twelve hours post-infection (hpi) the DNA was isolated by using DNeasy Blood and Tissue kit (QIAGEN) (as per manufacturer’s instructions). DNA was analyzed as described in detail in De Oliveira et al. [37] with minor modifications. PCR followed by gel electrophoresis was performed to confirm the presence of viral DNA. The primers GFP forward 5′-TGACCCTGAAGTTCATCTGCACCA-3′ and GFP reverse 5′-AACTCCAGCAGGACCATGTGAT-3′ were used. Then qPCR was performed by using Applied Biosystems™ SYBR™ Green PCR Master Mix on 96-well MicroAmp™ Optical 96-Well Reaction Plate. Also, a tenfold serial dilution was performed up to the 10^−5^ dilution to determine the standard curve equation.

### 2.13. Bioinformatic Analyses

The crystallized structure of glycoprotein D (4MYV) was obtained from Protein Data Bank (https://www.rcsb.org/structure/4MYV, accessed on 23 May 2022). The model of EGCG-S was prepared by Vega ZZ (https://www.ddl.unimi.it/cms/index.php?Software_projects:VEGA_ZZ, accessed on 21 May 2022). CASTp was used to help predict binding pockets for the glycoprotein D (http://sts.bioe.uic.edu/castp/index.html?4myv, accessed on 23 May 2022). The ribbon model was obtained from BIOVIA Discovery Studio (3ds.com, accessed on 11 June 2022) and AutoDock Vina (vina.scripps.edu, accessed on 10 June 2022) was used to model the interaction between EGCG-S and glycoprotein D. Binding affinity of −7.0 kcal/mol or stronger was used as the standard for what is considered acceptable [45,46,47]. Output files were examined in AutoDockTools where they were later opened to be observed at a better resolution with BIOVIA Discovery Studio. Two-dimensional images of receptor–ligand interactions were observed through BIOVIA Discovery Studio.

### 2.14. Statistical Analyses

Statistical analyses were performed by using Microsoft Excel. Statistical differences in the data from attachment and penetration assays and HSV-2 DNA copy number measured by qPCR were analyzed by using Student’s *t*-test. A minimum of three to five replicates were performed in each of the conditions.

## 3. Results

### 3.1. No Toxic Effect of EGCG and EGCG-S on Vero Cell Viability and Proliferation

**Figure 1:**
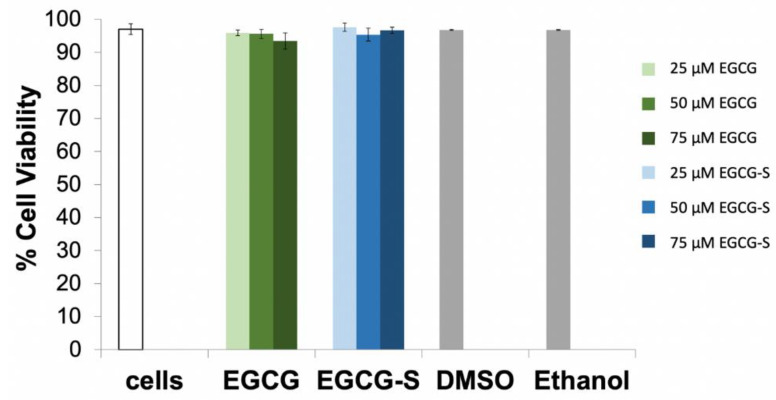
Cell viability assay with EGCG and EGCG-S. Vero cells were cultured in 6-well plates and treated with 25, 50, and 75 µM concentrations of EGCG and EGCG-S, respectively for one hour. DMSO and ethanol were used to determine their effect on Vero cells. The percentage of Vero cell viability was determined by using Trypan blue.

A determinant of cytotoxicity of EGCG or EGCG-S to Vero cells is to quantitatively determine the effect on cell viability by using the Trypan blue assay. The effect on cell proliferation was measured with the WST-1 reagent after 24 h of treatment (Roche Diagnostics, Indianapolis, IN, USA). The percentage of cellular growth was calculated relative to untreated Vero cells as the control (100% growth). Cell viability assays (Figure 1) show that concentrations up to 75 μM of EGCG and EGCG-S do not have major effects on the viability of Vero cells. Cell viability assays indicate that there is a slight decrease in the number of viable cells with increasing concentrations of EGCG and EGCG-S (all above 90% viability). The study also indicated that there were no observed effects of dissolving EGCG or EGCG-S in either ethanol or DMSO; therefore, further studies were continued by using only DMSO as the solvent at the following percentages (0.5%, 1%, and 1.5%, in 25, 50, and 75 μM concentrations, respectively).

**Figure 2:**
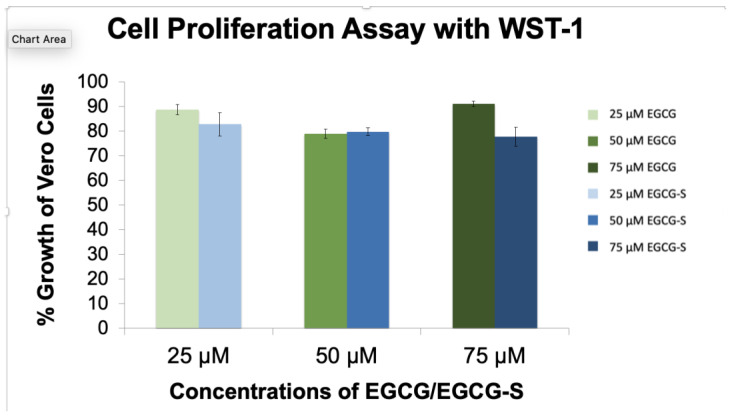
Cell proliferation assay with EGCG and EGCG-S. Cells were cultured in 96-well plates and treated 24 h later with 25, 50, and 75 µM concentrations of EGCG and EGCG-S, respectively for one hour. The percentage of cellular proliferation was determined with the colorimetric assay, WST-1, 24 h post-treatment. The absorbance was read at a wavelength of 450 nm. Means are shown with standard deviation (4 replicates).

The cell proliferation assays (Figure 2) confirm that EGCG and EGCG-S do not have major effects on the proliferation of Vero cells. The cells treated with EGCG or EGCG-S resulted in a minimum of 80% cell proliferation compared to the untreated Vero cells (100%). This indicated that these compounds do not have a toxic effect on Vero cells at the concentrations tested.

### 3.2. Infection of Vero Cells with Treated HSV-2 Reduces Cytopathic Effects

**Figure 3:**
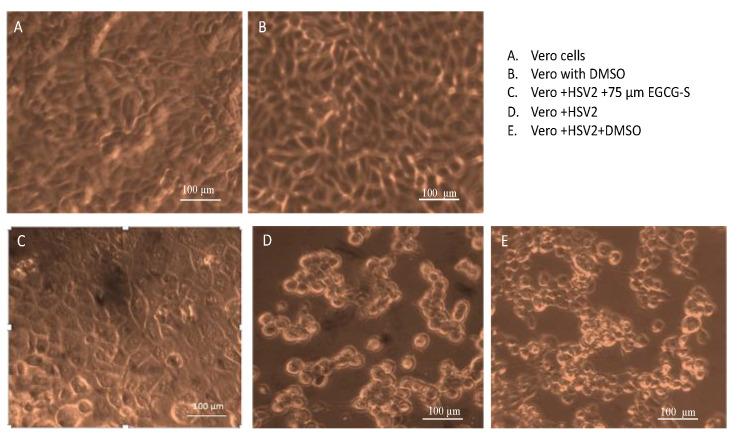
The morphological changes of Vero cells at different conditions. Vero cell monolayers were infected with EGCG-S treated or untreated HSV-2. Photographs were taken 48 h post-infection. (**A**) Uninfected and untreated Vero cell monolayers; (**B**) Vero cell monolayers treated with DMSO (1.5% concentration); (**C**) Vero cell monolayers infected with 75 µM EGCG-S treated HSV-2; (**D**) Vero cell monolayer infected with HSV-2; and (**E**) Vero cell monolayer treated with DMSO and infected with HSV-2.

The Vero cell monolayers were infected with HSV-2 for 48 h and observed with an inverted microscope. Treatment with 1.5% DMSO did not indicate cytotoxic effects (Figure 3B). There was no evidence of cytopathic effect in Vero cells infected with HSV-2 treated with 75 µM EGCG-S (Figure 3C) as compared to Vero cells infected with HSV-2 and the untreated Vero cell monolayer (Figure 3A). Vero cells infected with HSV-2 are rounded and lifted (Figure 3D,E). Treatment with DMSO did not affect the infectivity of HSV-2 (Figure 3E). However, treatment of HSV-2 with EGCG-S (Figure 3C) did not result in distinct changes indicative of cytopathic effect of Vero cells, as compared to uninfected Vero cells (Figure 3A), indicating inhibition of infection by EGCG-S.

### 3.3. EGCG and EGCG-S Inhibit HSV-2 Plaque Formation

To determine quantitatively how EGCG and EGCG-S affect viral replication, the plaque assay was carried out to determine the titer of the virus and the effect of treatment with EGCG and EGCG-S. EGCG or EGCG-S at 75 µM concentration was used to treat the viral lysate for 1 h. After treatment of the virus, Vero cell monolayers were infected with virus dilutions ranging from 10^−3^ to 10^−7^. The results are from the mean and SD of four experiments as shown in Table 1. The plaque-forming unit (PFU)/mL for the controls are 1.9, 1.10, 1.40, and 1.60 × 10^6^, and the titers for 75 µM EGCG-treated HSV-2 were 430, 350, 120, and 200 PFU/mL, respectively (*p* = 0.003; Student’s *t*-test). The mean and standard deviation of four independent experiments for percentage of inhibition is 99.981 ± 0.010. For the 75-µM EGCG-S treated HSV-2, the PFUs were 250, 350, 280, and 320 PFU/mL, respectively (*p* = 0.003; Student’s *t*-test). The mean and standard deviation of the percentage of inhibition is 99.979 ± 0.008. The results suggest that both EGCG and EGCG-S at a concentration of 75 µM can inhibit the plaque formation near completion.

### 3.4. EGCG and EGCG-S Affect the Attachment of HSV-2

**Figure 4:**
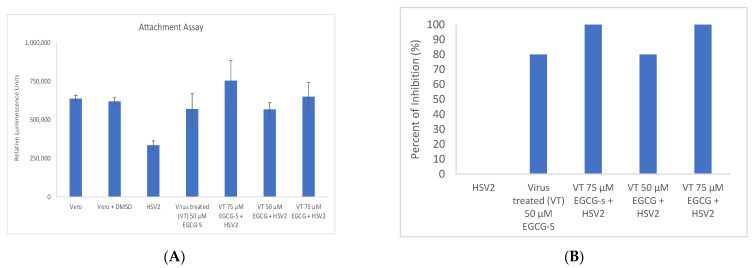
ToxGlo™ attachment assay with Vero cells infected at 4 °C with HSV-2 (MOI = 0.1) pretreated for one hour with 50 µM EGCG-S, 75 µM EGCG-S, 50 µM EGCG, and 75 µM EGCG, and Vero cells infected with untreated HSV-2 as control at 4 °C. (**A**) The antiviral effects of EGCG-S on the binding step of the viral cycle are reported. Cell viability was measured by the luminescence of Vero cells following infection with HSV-2 treated with 50 and 75 µM concentrations of EGCG and EGCG-S, respectively. (**B**) The results of the percent inhibition resulting from the treatment of HSV-2 with 50 and 75 µM concentrations of EGCG and EGCG-S are reported. Seventy-five µM concentrations of EGCG and EGCG-S, respectively, resulted in 100% inhibition of attachment to Vero cells.

To elucidate the potential mechanism for EGCG and EGCG-S on HSV-2 infection, experiments were carried out to determine if EGCG and EGCG-S affect the attachment of HSV-2 to Vero cells. The ToxGlo™ system was adapted to determine the effect on attachment to Vero cells and found that pretreatment of HSV-2 with 50 µM EGCG-S, 75 µM EGCG-S, 50 µM EGCG and 75 µM EGCG significantly reduced attachment of HSV-2 virions (*p* < 0.01; Student’s *t*-test) (Figure 4A). Treatment of HSV-2 with 75 µM of both EGCG-S and EGCG resulted in near 100% inhibition (Figure 4B). We conclude that both EGCG-S and EGCG inhibit the attachment of HSV-2 to Vero cells.

### 3.5. EGCG and EGCG-S Reduce HSV-2 Penetration in Vero Cells

**Figure 5:**
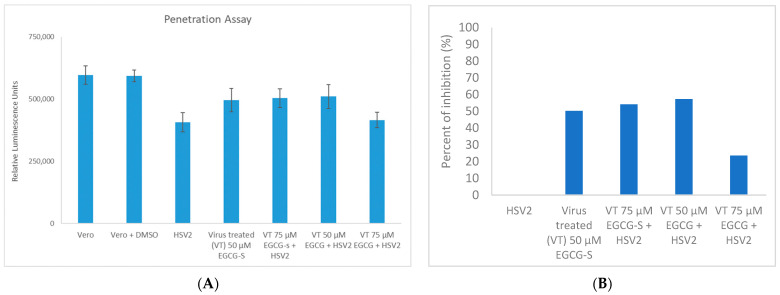
ToxGlo™ penetration assay with Vero cell monolayers infected with HSV-2 (MOI = 0.1) in the presence of 50 µM EGCG-S, 75 µM EGCG-S, 50 µM EGCG, and 75 µM EGCG, respectively. Unadsorbed virus was deactivated with PBS (pH 3) for 1 min and PBS (pH 11) to neutralize the acidic pH. Treatment of HSV-2 with 50 µM and 75 µM concentrations of EGCG and EGCG-S had some effect on the penetration step of the infection process. (**A**) Cell viability as measured by luminescence 48 hpi indicates that treatment of virions with 50 and 75 µM concentrations of EGCG and EGCG-S increased cell viability compared with untreated HSV-2. (**B**) The percent inhibition of penetration stage of HSV-2 infection when virions were treated with 50 and 75 µM concentrations of EGCG and EGCG-S is reported.

Vero cell monolayers were exposed to HSV-2 to allow attachment, but not penetration (as described in the Methods section) [43,44]. Cells were subsequently treated with 50 µM EGCG-S, 75 µM EGCG-S, 50 µM EGCG, and 75 µM EGCG, respectively, and then the virus was allowed to penetrate the cells. These results (Figure 5A,B) suggest that 50 µM EGCG-S, 75 µM EGCG-S, inhibit penetration 50.3% and 54.1%, respectively, and 50 µM EGCG, 75 µM EGCG inhibit penetration of HSV-2 57.3% and 23.6% respectively in Vero cell monolayers (*p* < 0.05; Student’s *t*-test).

### 3.6. Confocal Microscopic Observation

**Figure 6:**
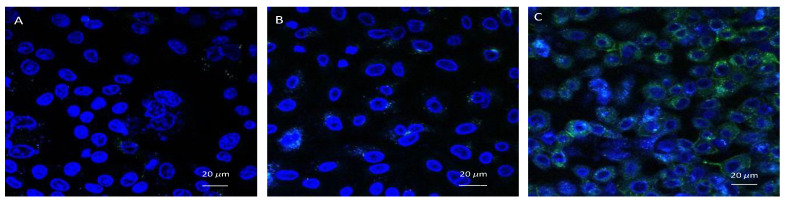
Confocal microscopic image (400Å~) of (**A**) uninfected Vero cells; (**B**) 12 h post-infection observation of EGCG-S-treated HSV-2 in Vero cells; and (**C**) 12 h post-infection observation of non- treated HSV-2 in Vero cells.

HSV-2 VP26-GFP contains a fusion protein of VP26 and green fluorescent protein (GFP) [40]. The expression of GFP indicates successful infection by HSV-2 (Figure 6C). If the GFP is not expressed, then the treatment with EGCG-S inhibited the successful infection of Vero cell monolayers by HSV-2. The HSV-2 infected cells express the GFP and serve as the untreated HSV-2 infected control (Figure 6C). When 75 µM EGCG-S treated HSV-2 infected the Vero cell monolayer, only slight expression of GFP was observed (Figure 6B). The GFP-expression in the image of the Vero cells infected with EGCG-S-treated HSV-2 is very similar to the image of the uninfected Vero cell monolayer (Figure 6A). This illustrated that 75 µM EGCG-S treated HSV-2 inhibited viral replication. VP26-GFP is expressed late in the replication cycle of untreated HSV-2 and GFP expression is pronounced in Figure 6C. Confocal microscopy indicates that the treatment of virions with EGCG-S inhibits the infection of Vero cells.

### 3.7. qPCR Quantitative Analysis of EGCG-S Treated or Non-Treated HSV-2

**Figure 7:**
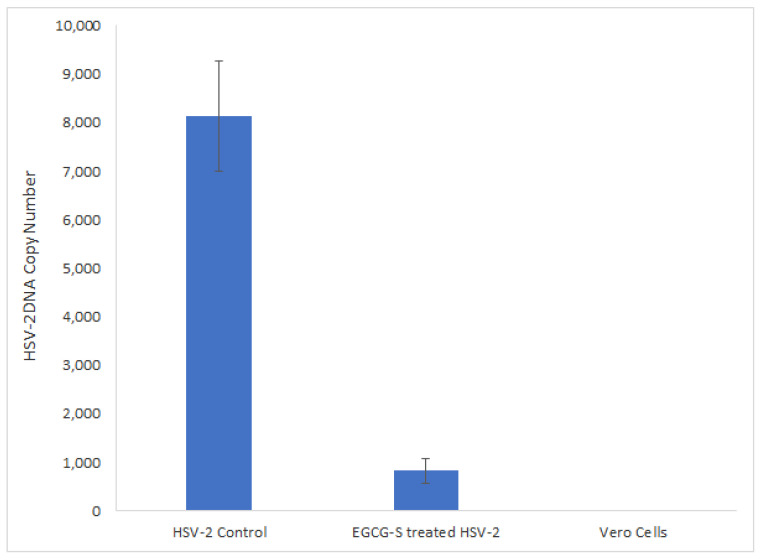
Real-time PCR (qPCR) analysis of HSV-2 DNA extracted from Vero cells infected with untreated HSV-2 and HSV-2 pretreated with 75 µM EGCG-S. The viral DNA copy number was determined by using a standard curve. Statistical significance was determined by using a paired Student’s *t*-test.

The effect of treatment of HSV-2 virions with EGCG-S was also quantitively determined by measuring the viral DNA yields by using a qPCR-based assay. The viral DNA concentrations in HSV-2 cultures treated with 75 µM EGCG-S and in untreated virus control were calculated and shown in Figure 7. The copy number of HSV-2 DNA showed a prominent decline in cultures infected with EGCG-S pre-treated HSV-2 compared to those of untreated virus controls. This assay demonstrated ~90% inhibition of HSV-2 proliferation when the HSV-2 virions were treated with EGCG-S prior to infection of Vero cells (*p* < 0.0001). No viral DNA was detected in non-infected cells which served as the negative control.

### 3.8. Bioinformatic Analysis of EGCG-S Binding to Glycoprotein D

**Figure 8:**
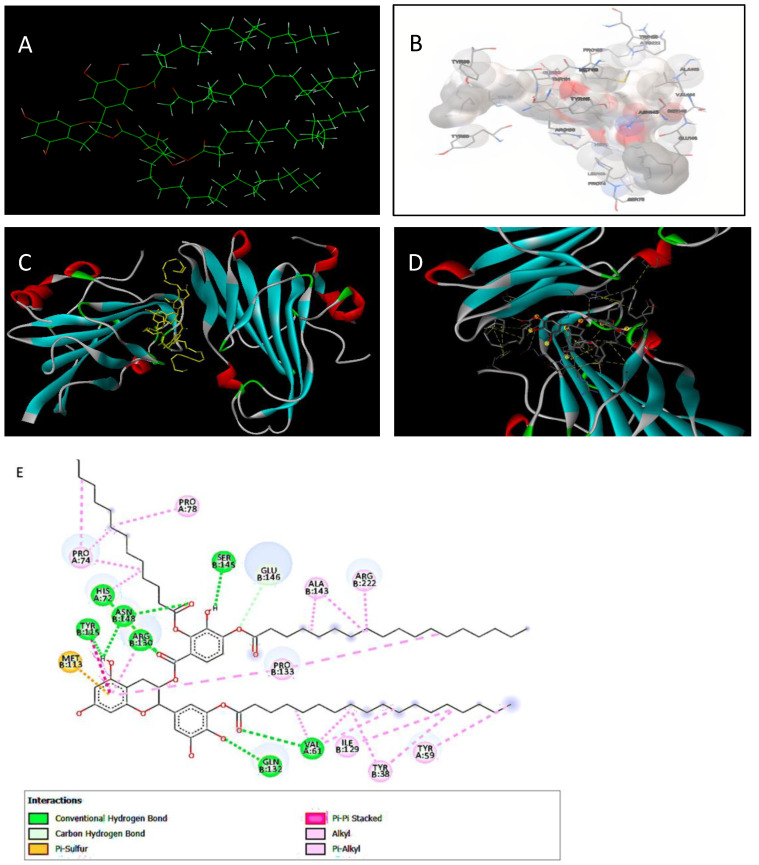
Bioinformatic analysis of the interaction of HSV-2 glycoprotein D (gD) and EGCG-S. (**A**) 3D Structure of EGCG-S in VegaZZ; (**B**) Predicted binding interaction between EGCG-S and glycoprotein D based on AutoDock Vina analysis; (**C**) Ribbon model of binding between glycoprotein D and EGCG-S; (**D**) Enhanced ribbon image of binding between glycoprotein D and EGCG-S; (**E**) Two-dimensional diagram of receptor-ligand interactions including conventional hydrogen bonds (green), van der Waals (light green), pi-sulfur (yellow), pi–pi stacked (dark pink), alkyl and pi-alkyl (pink).

The 3D structure of EGCG-S (Figure 8A), the predicted bonding interaction between EGCG-S and glycoprotein D (Figure 8B), and the Ribbon model of binding between glycoprotein D and EGCG-S (Figure 8C,D) and receptor-ligand interactions (Figure 8E) are illustrated and summarized. EGCG-S can bind to glycoprotein D with a binding affinity of −7.2 kcal/mol (Figure 8E). EGCG-S forms seven conventional hydrogen bonds (Chain A: 61 VAL, 72 HIS, Chain B: 115 TYR, 130 ARG, 132 GLN, 145 SER, 148 ASN), one carbon-hydrogen bond (Chain B: 146 GLU), one pi-sulfur bond (Chain B: 113 MET), and eight alkyl/pi-alkyl bonds (Chain A: 59 TYR, 74 PRO, 78 PRO, Chain B: 38 TYR, 129 ILE, 133 PRO, 143 ALA, 222 ARG). The main regions of binding are the middle benzenediol and the palmitates. The pi-sulfur bond helps with protein stabilization and folding is one of the more important aromatic ring configurations [46,47]. The bioinformatic analysis indicated that EGCG-S has a high binding affinity to glycoprotein D and suggested a possible mechanism of EGCG-S to inhibit HSV-2 adsorption on Vero cells.

## 4. Discussion

HSV-2 infections are lifelong infections and result in painful blisters and lesions at the site of infection. The lytic infection cycle of HSV-2 begins with the process of adsorption. Natural products are a source of potential anti-HSV-2 agents. The goal of our study was to investigate the efficacy and modes of action of epigallocatechin gallate (EGCG) and a more stable structural analog EGCG-stearate (EGCG-S) against HSV-2 in cultured Vero cells, a continuous cell line used to cultivate many different viruses and to determine the antiviral potential of several reagents [27,44,48,49,50]. Previous research reported that EGCG and lipophilic EGCG inhibit the infection of a variety of RNA and DNA viruses, enveloped and non-enveloped [18,21,22,23,24,25,26,48,49]. EGCG lipid esters were reported to be 24-fold more effective than EGCG as inhibitors and activators of influenza virus [51]. Therefore, lipophilic EGCG is a good candidate for a topical antiviral agent. Additionally, lipophilic EGCG was found to inactivate HSV-1 [36,37,38]. The cytotoxicity study showed that EGCG and EGCG-S were not cytotoxic to Vero cells at concentrations tested (Figure 1 and Figure 2). We previously reported that EGCG and EGCG-S were not cytotoxic to cultured A549 human lung fibroblast cells at concentrations up to 75 µM [38]. We demonstrated that EGCG and EGCG-S inhibit the infection of HSV-2 in cultured Vero cells at a concentration of 75 µM with greater than 99.9% inhibition (Table 1). EGCG, however, is not stable and therefore has limited its therapeutic potential [33,34].

The lytic infection cycle of HSV-2 begins with the process of adsorption. HSV is required to attach itself to host cells and fuse the envelope to allow for cellular uptake. Previous research demonstrated that EGCG formed complexes with purified HSV-1 glycoproteins B and D [27]. The virion first attaches to, then fuses with, a host cell plasma membrane. Glycoproteins B and C (gB and gC) mediate the loose attachment of HSV virions to heparan sulfate proteoglycans on host cells. High-affinity binding of gD results in formation of a fusion complex consisting of gB, gH, and gL. This fusion of complex and cellular receptors results in membrane fusion followed by the entry of virions via endocytosis. Both attachment and penetration take place when viral glycoproteins bind to cellular receptors on the plasma membrane of the host cell [48,49]. Treatment of virions with EGCG and EGCG-S was shown to affect the attachment stage and to a lesser extent, the penetration stage in cultured Vero cells (Figure 4 and Figure 5). This finding is consistent with a previous study in which a lipophilic form of EGCG, palmitoyl EGCG (p-EGCG), inhibited the attachment of HSV-1 in cultured Vero cells [37]. Modification of EGCG with palmitate increased the effectiveness of EGCG as an antiviral agent. Results of cell viability and cell proliferation assays indicated that p-EGCG is not toxic to cultured Vero cells [37,52], indicating that it can be applied topically. The reduced amplification of viral DNA (Figure 7) complemented and strengthened our findings that EGCG-S inhibited the early stage (attachment) of infection of HSV-2. A previous study reported that EGCG could form water-soluble complexes with biotin [53]. Despite this report of non-specific binding, EGCG-S has been safely used as a topical agent in patients with recurring HSV-1 infections [39]. A major advantage of EGCG as a potential antiviral agent is that it is non-toxic and can be consumed or applied topically [54].

In silico analysis demonstrated a high binding affinity of EGCG-S for glycoprotein D, rendering gD unavailable to form the fusion complex between the virion and cell membrane. This prevents entry of HSV-2 into the infected cell via endocytosis, confirming the effect on attachment.

## 5. Conclusions

Our results demonstrate that treatment of HSV-2 virions with EGCG and EGCG-S, respectively, at a concentration of 75 µM showed greater than 99.9% inhibition by inhibiting the attachment of HSV-2 virions to host cells. The bioinformatic analysis demonstrated the high-affinity binding of EGCG-S to gD. EGCG-S could be developed as a component of a topical agent to treat lesions of HSV-2 infections due to its safety and effectiveness. This could contribute to reducing the spread of HSV-2 infections.

## Figures and Tables

**Table 1:** Viral titers in response to 75 µM EGCG and 75 µM EGCG-S treatments of HSV-2 virions.

Plaque Assay	Untreated HSV-2 (PFU/mL)	75 μM EGCG (PFU/mL)	% of Inhibition	Mean & STDEV	75 μM EGCG-S (PFU/mL)	% of Inhibition	Mean & STDEV
# 1	1.90 × 10^6^	430	99.977	99.981 ± 0.0104	250	99.987	99.979 ± 0.008
# 2	1.10 × 10^6^	350	99.968		350	99.968	
# 3	1.40 × 10^6^	120	99.991		280	99.980	
# 4	1.60 × 10^6^	200	99.987		320	99.980	

## Data Availability

The datasets supporting the conclusions of this article are included within the article.
